# Cabazitaxel‐Loaded Thermosensitive Hydrogel System for Suppressed Orthotopic Colorectal Cancer and Liver Metastasis

**DOI:** 10.1002/advs.202404800

**Published:** 2024-06-27

**Authors:** Yu Chen, Liqun Dai, Kun Shi, Meng Pan, Liping Yuan, Zhiyong Qian

**Affiliations:** ^1^ Department of Biotherapy Cancer Center and State Key Laboratory of Biotherapy West China Hospital Sichuan University Chengdu 610041 China

**Keywords:** colorectal cancer, CTX/PLEL, liver metastasis, orthotopic mouse model, thermosensitive

## Abstract

The treatment of colorectal cancer is always a major challenge in the field of cancer research. The number of estimated new cases of colorectal cancer worldwide in 2020 is 1 148 515, and the estimated number of deaths is 576 858, revealing that mortality accounted for approximately half of the disease incidence. The development of new drugs and strategies for colorectal cancer treatment is urgently needed. Thermosensitive injectable hydrogel PDLLA‐PEG‐PDLLA (PLEL) loaded with cabazitaxel (CTX) is used to explore its anti‐tumor effect on mice with orthotopic colorectal cancer. CTX/PLEL is characterized by a solution state at room temperature and a hydrogel state at physiologic temperature. The excipients MPEG‐PCL and PDLLA‐PEG‐PDLLA have good biocompatibility and biodegradability. The simple material synthesis and preparation process renders this system cost‐effective and more conducive to clinical transformation. An orthotopic colorectal cancer model is established by transplantation subcutaneous tumors onto the cecum of mice. According to the results of experiments in vivo, CTX/PLEL significantly inhibits orthotopic colorectal cancer and liver metastasis in mice. The results indicate that CTX/PLEL nanoparticle preparations have high security and excellent anti‐tumor effects, and have great application potential in colorectal cancer therapy.

## Graphical abstract

This work uses temperature‐responsive injectable hydrogel PLEL loaded with cabazitaxel (CTX) to explore its therapeutic effect on mice with orthotopic colorectal cancer. CTX/PLEL presents a solution state at room temperature and a hydrogel state at physiological temperature. In orthotopic mouse model of colorectal cancer, CTX/PLEL significantly inhibits colorectal cancer and liver metastasis.

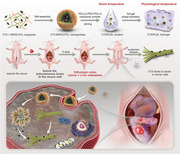

## 1 Introduction

The treatment of colorectal cancer has always been a major challenge in the field of cancer research. The number of estimated new cases of colorectal cancer worldwide in 2020 was 1 148 515, and the estimated number of deaths was 576 858, revealing that mortality accounted for approximately half of the disease incidence. The incidence and mortality of colorectal cancer have increased in China in recent years, which may be related to dietary patterns, obesity, and lifestyle factors.^[^ ^1^, ^2^, ^3^ ^]^ Metastasis is the main cause of death in patients with colorectal cancer. The most common site of colorectal cancer metastasis is the liver, followed by the lungs, peritoneum, and distant lymph nodes.^[^ ^4^, ^5^ ^]^

At present, the treatment of colorectal cancer mainly includes surgery, chemotherapy, and immunotherapy.^[^ ^6^, ^7^ ^]^ Surgery is the primary treatment for early and late‐stage colorectal cancer. However, most patients with distal metastasis and recurrence cannot use this method, and 30%−50% of patients have recurrence after surgery, usually manifested as metastasis.^[^ ^8^, ^9^ ^]^ Patients with colorectal cancer have lower degree of vascularization of the descending colon and rectum, which is not conducive to the delivery of chemotherapy drugs. This results in weaker therapeutic effect and higher systemic toxicity. In addition, drug resistance in advanced patients seriously affects the efficacy of chemotherapy.^[^ ^10^, ^11^, ^12^ ^]^ Immune checkpoint blocking therapy (ICB) has lower systemic toxicity than chemotherapy. Immune checkpoint inhibitors Pembrolizumab, Nivolumab, and Ipilimumab have been approved for patients with microsatellite instability—high (MSI‐H, a hypermutable phenotype) refractory metastatic colorectal cancer. But 85% of colorectal cancer patients and 96% of metastatic colorectal cancer patients do not have MSI‐H. ICB therapy is ineffective in these patients.^[^ ^7^, ^13^ ^]^ The development of new drugs and strategies for colorectal cancer treatment is urgently needed.

Local treatment can overcome the problems of low targeting ability and high systemic toxicity of intravenous administration in patients with colorectal cancer. Increasing the concentration and duration of the drug at the tumor site is the key to improve the efficacy of the drug^[^ ^14^ ^]^ Our group constructed a thermosensitive hydrogel PDLLA‐PEG‐PDLLA (PLEL), which has a simple production process, controllable molecular weight and block ratio. It is characterized by a solution state at room temperature and a hydrogel state at physiologic temperature.^[^ ^14^ ^]^ As a thermosensitive hydrogel with the advantages of good biocompatibility and biodegradability, PLEL can be used to load chemotherapy drugs for local administration in patients with colorectal cancer

Taxanes are a class of small‐molecule chemotherapeutic drugs. Their unique mechanism involves binding to the β‐subunit of tubulin to inhibit the depolymerization of microtubules during mitosis, thereby promoting cancer cell apoptosis.^[^ ^15^, ^16^, ^17^, ^18^, ^19^, ^20^, ^21^ ^]^ Paclitaxel (PTX) and Docetaxel (DTX) are first‐generation taxanes. Taxol (PTX), Taxotere (DTX), and Abraxane (PTX) have been successful in the field of oncology therapy.^[^ ^22^, ^23^, ^24^ ^]^ Second‐generation taxane, cabazitaxel (CTX, Jevtana; Sanofi‐Aventis) was approved by the FDA in June 2010 for patients with metastatic castration‐resistant prostate cancer that has progressed after docetaxel treatment.^[^ ^16^ ^]^ As a derivative of PTX, the unique chemical structure of CTX gives it the advantage of anti‐tumor efficacy superior to PTX and DTX, as well as a low affinity to p‐glycoproteins.^[^ ^25^, ^26^, ^27^, ^28^, ^29^, ^30^, ^31^, ^32^, ^33^ ^]^ However, Jevtana must be diluted twice before use (no more than 30 min between the two dilutions) and then completed intravenously within 8 h of the second dilution (room temperature, 24 h for 2–8 °C).^[^ ^34^ ^]^ The complicated operation and limited stability of Jevtana affect its application for the treatment of cancer. CTX loaded with thermosensitive injectable hydrogel PLEL can reduce systemic toxicity, improve the stability of the preparation, and enhance the therapeutic effect.

**Scheme 1:**
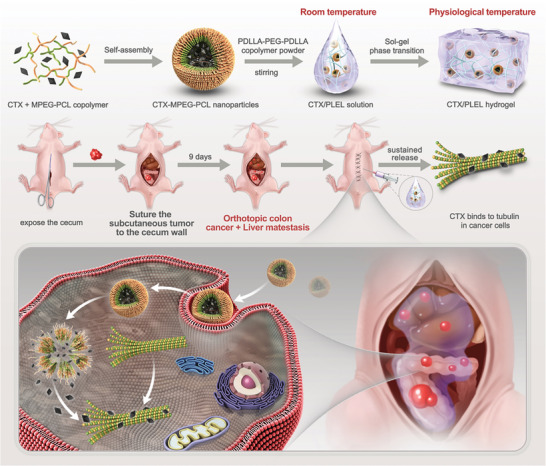
Schematic illustration of preparation and in vivo antitumor effect of CTX/PLEL.

Based on the above research, we constructed an orthotopic model of colorectal cancer in mouse with a high rate of tumorigenesis, which can represent both orthotopic colorectal cancer and liver metastasis. At present, there are few reports of CTX loaded with hydrogel. We used thermosensitive injectable hydrogel PLEL loaded with CTX to explore its therapeutic effect on mice with orthotopic colorectal cancer (**Scheme** **1**). CTX/PLEL presents a solution state at room temperature and a hydrogel state at physiological temperature. Because of the hydrophobicity of CTX, MPEG‐PCL was used to prepare CTX‐MPEG‐PCL nanoparticles by the emulsification solvent volatilization method. The excipients MPEG‐PCL and PDLLA‐PEG‐PDLLA have good biocompatibility and biodegradability. The simple material synthesis and preparation process renders this system cost‐effective and more conducive to clinical transformation. We established an orthotopic colorectal cancer model by transplantation subcutaneous tumors onto the cecum of mice. Compared with the classical orthotopic model of inoculating cells into the cecum wall, this method is more likely to reflect tumor metastasis and has a higher tumorigenesis rate.

## 2 Results and Discussion

### 2.1 Preparation and Thermosensitive Sol‐Gel Phase Transition of CTX/PLEL

According to previous research, we synthesized MPEG_2000_‐PCL_10000_ and PDLLA_1500_‐PEG_1500_‐PDLLA_1500_ copolymers by ring‐opening polymerization to prepare cabazitaxel‐loaded thermosensitive hydrogel system.^[^ ^14^, ^35^, ^36^, ^37^, ^38^, ^39^ ^]^ The ^1^H‐NMR spectra of MPEG_2000_‐PCL_10000_ and PDLLA_1500_‐PEG_1500_‐PDLLA_1500_ copolymers are shown in Figures S1 and S2 (Supporting Information). The analytical results of chemical shift and corresponding group are listed in Tables S1 and S2 (Supporting Information). These results indicated the successful synthesis of MPEG_2000_‐PCL_10000_ and PDLLA_1500_‐PEG_1500_‐PDLLA_1500_ copolymers.

**Figure 1:**
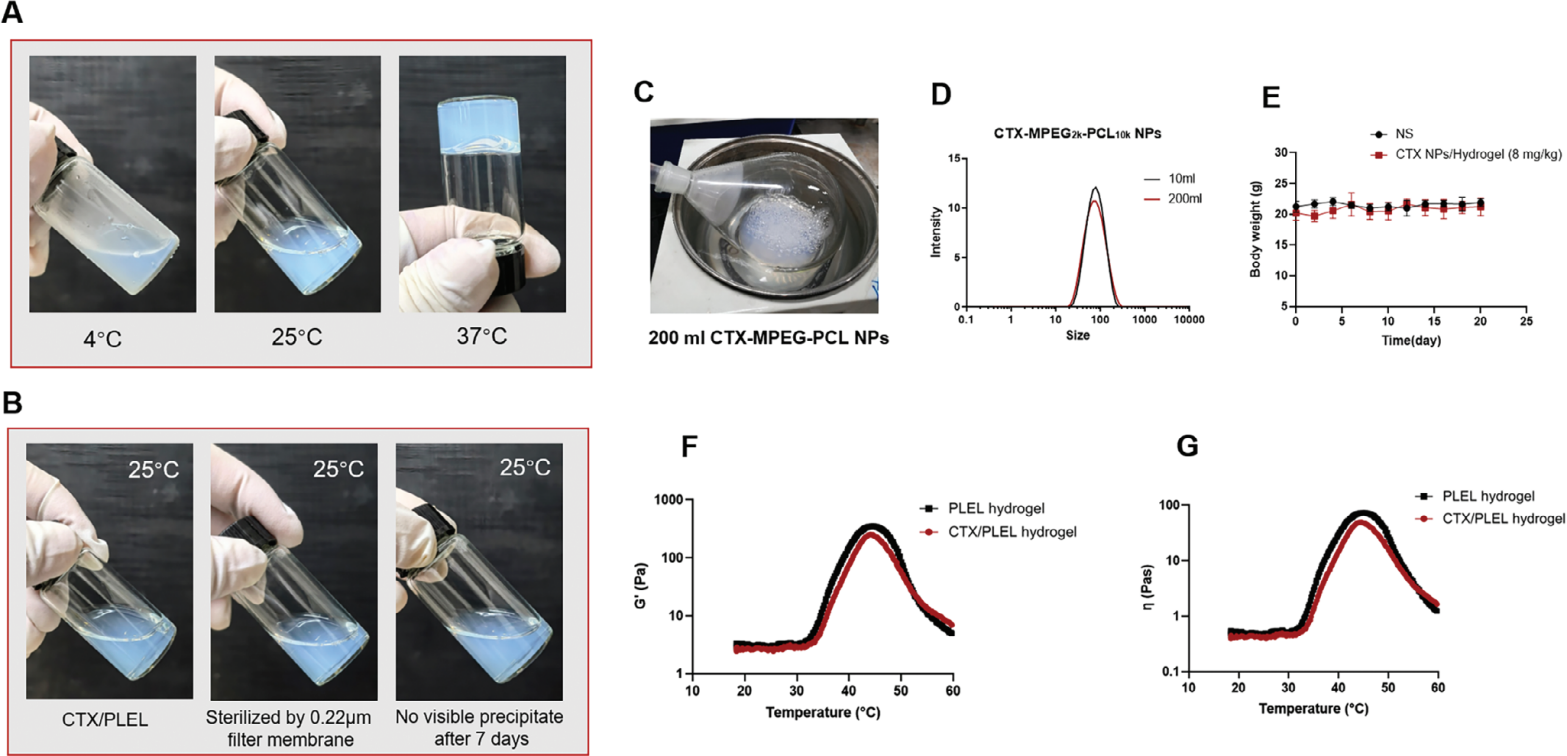
Characterization of CTX/PLEL. A) Thermosensitive sol‐gel phase transition of CTX/PLEL. B) The stability of CTX/PLEL. C) The rotary evaporation for preparing 200 mL CTX‐MPEG‐PCL in the laboratory. D) Particle size distribution of CTX‐MPEG‐PCL in 10 and 200 mL systems. E) The average body weight change curves of healthy balb/c mice in each group after intraperitoneal injection. (n = 4). F) Temperature‐dependence of storage modulus (G′) for the CTX loaded PLEL hydrogel and PLEL hydrogel (15 wt.%). G) Temperature‐dependence of viscosity (η) for the CTX loaded PLEL hydrogel and PLEL hydrogel (15 wt.%).

The complicated operation and limited stability of Jevtana affect its application for the treatment of cancer. In addition, there are many possible factors affecting the progress of CTX formulation research, such as the difficulty, feasibility, and repeatability of mass production, and production cost.^[^ ^40^, ^41^, ^42^ ^]^ According to our previous research, the drug‐loading of CTX‐MPEG_2k_‐PCL_10k_ nanoparticles is 10.1% (drug feeding: 15%) with a particle size of 65–70 nm.^[^ ^38^ ^]^ **Figure** **1**C shows the rotary evaporation for preparing 200 mL CTX‐MPEG‐PCL in the laboratory. Figure 1D shows the nanoparticle size distributions of CTX‐MPEG‐PCL in 10 and 200 mL systems. These results indicate that large‐scale production of CTX‐MPEG‐PCL can be achieved under laboratory conditions.

The PDLLA‐PEG‐PDLLA (L_1500_‐E_1500_‐L_1500_, PLEL) polymer powder was added into the CTX‐MPEG‐PCL nanoparticle solution and mixed by magnetic stirring to obtain homogeneous PLEL polymer solution. The concentration of PLEL is 15 wt.%. The morphology of the PLEL micelles is shown in Figure S3 (Supporting Information). It can be seen that the nanoparticles have a uniform spherical distribution. As can be seen in Figure 1A, CTX/PLEL is a transparent and uniform solution at room temperature, which can be used for clinical injection. When the temperature is increased to 37 °C, the CTX/PLEL rapidly changes to a hydrogel state, which can be used to control the sustained release of the drug. Figure 1B shows that CTX/PLEL can be filtered through 0.22 µm syringe filter for bacteria removal, which is beneficial for industrial production and clinical applications. There was no visible precipitate in the solution after 7 days at room temperature, which was conducive to the clinical transformation of CTX/PLEL.^[^ ^43^, ^44^, ^45^, ^46^ ^]^

The phase transition behavior of CTX/PLEL was detected by rheological experiments. It can be seen from Figure S4A (Supporting Information) that PLEL solution completes reversible sol‐gel transition at 35 °C. The gel‐sol transition occurred at 46.4 °C, which was due to the structural damage of PLEL micelles and the break of PEG chains caused by high temperature. After the addition of small molecule drug, the sol‐gel transition temperature of CTX/PLEL increased to 35.8 °C (Figure S4B, Supporting Information). Figure 1F,G shows the curve of storage modulus (G′), and viscosity (η), it can be seen that there is no significant difference in temperature sensitivity and rheological properties between CTX/PLEL and blank PLEL. In addition, CTX/PLEL and blank PLEL can reach gelation rapidly at 37 °C with no significant difference in gelation time (t_gel_) (Figure S4C, Supporting Information). In general, the CTX/PLEL formulation can complete the reversible sol‐gel transition at 37 °C, which is conducive to control the sustained release of the drug.

The drug release of CTX/PLEL was quantitatively detected by high‐performance liquid chromatography (HPLC). Figure S5C,D (Supporting Information) shows the HPLC chromatogram of CTX released from PLEL hydrogel and CTX standards. It can be seen that CTX released from the hydrogel preparation has a stable structure. Figure S5A (Supporting Information) shows the in vitro release curve of CTX/PLEL in PBS buffer (pH 7.4) containing 0.1% w/v Tween 80 at 37 °C. At 48 h, the drug release rate of CTX/PLEL was approximately 11.4%, and at 96 h, the release rate was only approximately 16.8%. In conclusion, the loading of PLEL significantly slowed the in vitro drug release of CTX.

### 2.2 Safety Evaluation of CTX/PLEL In Vivo

To evaluate the in vivo safety of CTX/PLEL, we injected CTX/PLEL intraperitoneally into healthy balb/c mice at 8 mg kg^−1^. Mice in control group were intraperitoneally injected with normal saline. Orbital blood was taken on day 7 after administration for complete blood counts. Figure 1E shows the average body weight change curves of mice in each group after administration. Figure S6 (Supporting Information) shows the results of blood routine tests, including white blood cells (WBC), red blood cells (RBC), platelets (PLT), and hemoglobin (HGB). Compared with the control group, there was no significant difference in body weight and blood routine test results of mice injected with CTX/PLEL. These results indicate that CTX/PLEL has good biosafety and can be used in clinical treatment. Next, we will investigate the anti‐tumor effects of CTX/PLEL.

### 2.3 Antitumor Activity of CTX/PLEL In Vitro

**Figure 2:**
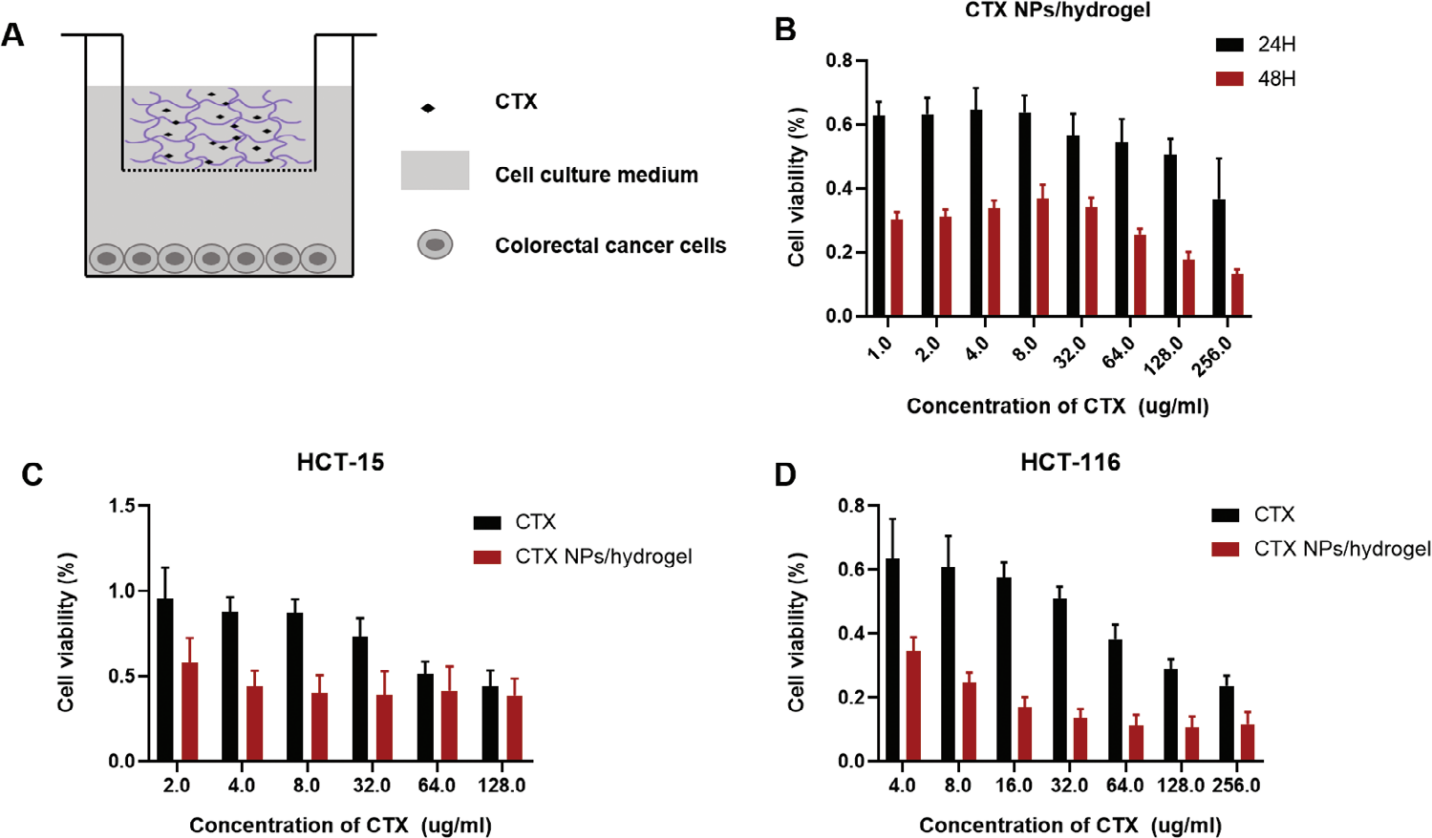
Cytotoxicity studies of CTX/PLEL on HCT‐15 cells and HCT‐116 cells measured by MTT assays.

The antitumor activity of CTX/PLEL in vitro was detected by MTT assay. The cell line used was human colorectal cancer cells (HCT‐15 and HCT‐116). As shown in **Figure** **2**A, when the hydrogel carrying CTX was added to the 96‐well plate, it completed the sol‐gel phase transition at 37 °C and slowly released the drug. In Figure 2B, CTX/PLEL exerted significant inhibitory effects on colorectal cancer cells, and the inhibitory degree increased with the increase of CTX concentration. The drug is released slowly over 48 h. Under the treatment of 128 ug mL^−1^ CTX, the cell viability of cells was 50.4% after 24 h of co‐culture and decreased to 17.8% after 48 h of co‐culture. These results indicated that the inhibitory activity of the hydrogel on cells increased with the extension of the cell culture time, suggesting that the hydrogel has the effect of sustained drug release. From Figure 2C,D, we can see that the CTX nano‐preparation group has a stronger inhibitory effect on the two types of colorectal cancer cells compared with free CTX. These results indicate that the cytotoxicity of CTX to colorectal cancer is enhanced after loading with PLEL hydrogel, which further proves that PLEL has the effect of slow release of CTX.

### 2.4 Orthotopic Mouse Model of Colorectal Cancer

**Figure 3:**
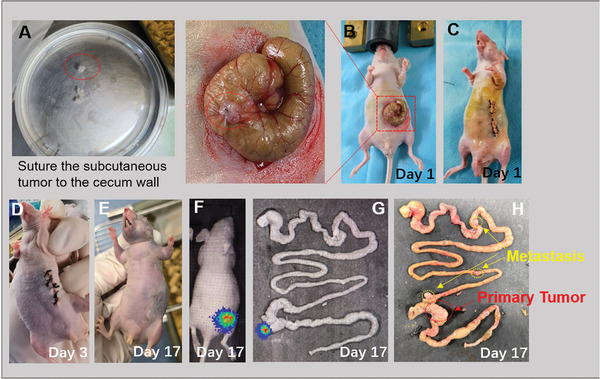
Establishment of an orthotopic colorectal tumor model in balb/c‐nu mice. The subcutaneous tumor was cut into small pieces of 2–3 mm and then sutured to the wall of the cecum with absorbable sutures. The mice were anesthetized by isoflurane throughout the operation and remained unconscious.

Orthotopic tumor models can better reflect the disease development of colorectal cancer patients than the subcutaneous tumor model.^[^ ^47^, ^48^, ^49^ ^]^ Due to differences in the tumor microenvironment, subcutaneous tumor models can hardly progress and metastasize. Metastasis is the leading cause of death in patients with colorectal cancer.^[^ ^50^, ^51^ ^]^ We constructed an orthotopic colorectal cancer model to investigate the antitumor activity of CTX/PLEL preparations. As shown in **Figure** **3**A, the subcutaneous tumor was cut into small pieces of 2–3 mm and then sutured to the wall of the cecum with absorbable sutures (Figure 3B). Finally, the abdomen skin of the mice was sutured (Figure 3C). The mice were anesthetized by isoflurane throughout the operation and remained unconscious. The abdominal skin of the mice did not develop infection and completely healed (Figure 3D,E). The shape of the tumor was observed by a fluorescence imaging system (IVIS Lumina Series III, PerkinElmer, USA) on day 17 after modeling. As shown in Figure 3F, there is strong bioluminescence in the cecum of mice. The mouse was sacrificed and the whole intestine was removed. Visible orthotopic tumors in the cecum and surrounding metastases can be seen (Figure 3H). The tumorigenesis rate of this orthotopic model is ≈80%.

### 2.5 Inhibition of Orthotopic Colorectal Cancer and Liver Metastasis by CTX/PLEL

**Figure 4:**
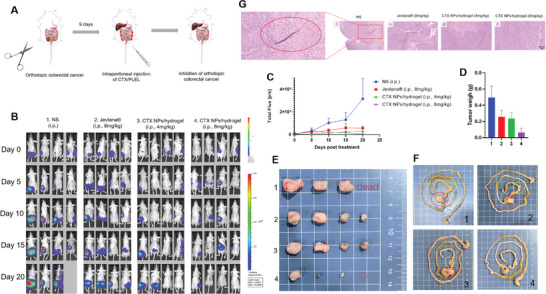
Inhibition of orthotopic colorectal cancer by CTX/PLEL. A) Establishment of orthotopic colorectal tumor models. The HCT‐116‐luc subcutaneous tumors were sutured to the mouse cecum wall. B) In vivo luciferase expression of orthotopic colorectal cancer in the whole mice. C) Tumor growth curves of orthotopic colorectal tumors. D) Final orthotopic tumor weights after 20 days. E) Images of the orthotopic tumors collected from various groups of mice at the end of the treatments (day 20). F) Representative intestine images from the mice after treatment. G) H&E‐staining of the orthotopic tumor tissues of each group after treatment. Scale bar:100 µm. (n = 4).

**Figure 5:**
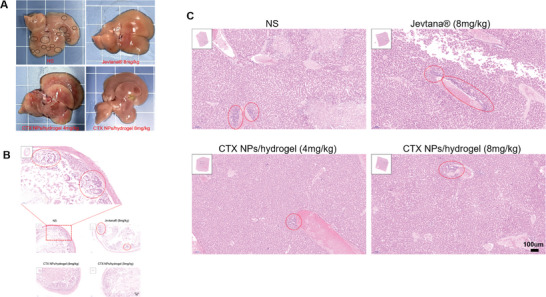
Inhibition of metastasis in orthotopic colorectal cancer by CTX/PLEL. A) Representative liver images from the mice after treatment. B) H&E‐staining of the intestine of each group after treatment. Scale bar:100 µm. C) H&E‐staining of the liver tissues of each group after treatment. Scale bar:100 µm.

We investigated the in vivo antitumor activity of CTX/PLEL using a colorectal orthotopic model (**Figure** **4**A). Mice bearing orthotopic tumors were randomLy divided into four groups (4 mice in each group) and intraperitoneally injected with normal saline (i.p.), Jevtana (i.p., 8 mg kg^−1^ of CTX), CTX /PLEL (i.p., 4 mg kg^−1^ of CTX), or CTX/PLEL (i.p., 8 mg kg^−1^ of CTX). The four groups were single injection. Luminescence intensities were recorded every five days. As can be seen from Figure 4B,C, CTX/PLEL (i.p., 8 mg kg^−1^ of CTX) more significantly blocked tumor growth than saline as control. On day 20, the mean bioluminescence intensity of tumor treated with CTX/PLEL (i.p., 8 mg kg^−1^ of CTX) was only 1.47% and 8.11% of those from the mice treated with saline and Jevtana (i.p., 8 mg kg^−1^ of CTX), respectively. This indicates that CTX/PLEL has better anti‐tumor efficacy than Jevtana at the same dose. In addition, after the dose was halved, the tumor growth rate of mice in the low‐dose CTX/PLEL group was lower than that in the high‐dose Jevtana group. It is suggested that CTX/PLEL preparation can reduce the dose of CTX and thus reduce the toxicity of the drug. These results are consistent with those of Figure 4E for tumors in vitro and in Figure 4D in terms of tumor weight. the mice treated with CTX/PLEL (i.p., 8 mg kg^−1^ of CTX) showed the lowest tumor weight, which was only 12.7% and 24.6% of the tumor weights from the mice treated with saline as control and Jevtana (i.p., 8 mg kg^−1^ of CTX), respectively (Figure 4D). Metastasis of colorectal cancer occurs most often in the liver, followed by the lungs, peritoneum, and distant lymph nodes.^[^ ^52^, ^53^ ^]^ The ascending colon, transverse colon, descending colon, and sigmoid colon are located between the cecum and rectum. Figure 4F and **Figure** **5**A showed that CTX/PLEL (8 mg kg^−1^ of CTX) inhibited the growth of metastatic tumors in the colon and liver in this orthotopic model.

**Figure 6:**
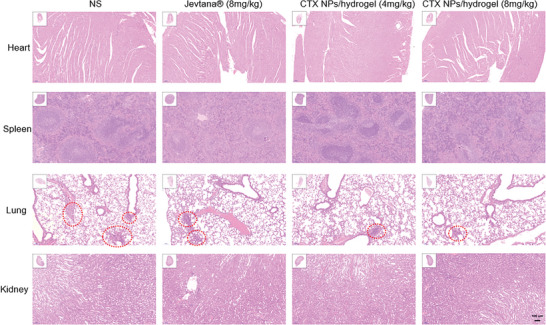
H&E‐staining of the heart, spleen, lung, and kidney tissues of each group after treatment. Scale bar:100 µm.

The clinical diagnosis of malignant tumor is mainly based on histopathological examination. Compared with normal cells, tumor cells have the characteristics of a larger nucleus, multinucleation, irregular cell morphology, and necrosis in the background of cancer. HE staining techniques use heme and paclitaxel to stain the nucleus and cytoplasm as blue‐purple and pink, and the progression of the tumor can be judged under the microscope.^[^ ^54^, ^55^, ^56^ ^]^ As can be seen from Figure 4G, the saline group contains a large number of tumor cells, and clear new blood vessels can be seen, which is a landmark feature of tumor tissue. Figure 5B,C and **Figure** **6** show obvious metastasis in intestine, liver, and lung, which were significantly inhibited in the CTX/PLEL group.

### 2.6 Anti‐Tumor Effects of CTX/PLEL in Subcutaneous Tumor Model of Colorectal Cancer

**Figure 7:**
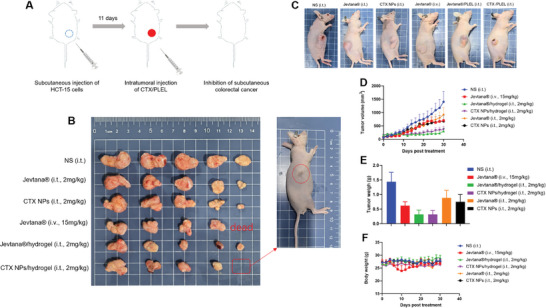
Anticancer effect in HCT‐15 human colorectal cancer subcutaneous tumor model. A) Establishment of a subcutaneous colorectal tumor model in balb/c‐nu mice. B) Images of the tumors collected from various groups of mice at the end of the treatments (day 30). C) Representative photos of mice bearing subcutaneous colorectal tumors (day 30). D) Tumor growth curves of various groups. E) Final tumor weights after 30 days. F) Changes in body weight of mice. (n = 5).

The in vivo antitumor activity of CTX/PLEL was further investigated by HCT‐15 human colorectal cancer subcutaneous tumor model. Colorectal cancer cells (HCT‐15) were injected subcutaneously (100 µL per mouse) at a density of 1 × 10^7^ cells mL^−1^ into the right back of female balb/c‐nu mice aged 6–8 weeks. When the tumor volumes reached to 100 mm^3^, the mice were randomLy divided into six groups (5 mice in each group) and injected with normal saline (i.t.), Jevtana (i.t., 2 mg kg^−1^ of CTX), CTX‐MPEG‐PCL NPs (i.t., 2 mg kg^−1^ of CTX), Jevtana (i.v., 15 mg kg^−1^ of CTX), Jevtana/PLEL (i.t., 2 mg kg^−1^ of CTX), or CTX/PLEL (i.t., 2 mg kg^−1^ of CTX). The intratumor groups were single injections, and the intravenous injection group was given on days 0,3, and 6 (**Figure** **7**A). The body weight and tumor volumes of the mice were recorded every other day. According to the tumor growth curve after administration (Figure 7D), CTX/PLEL (i.t.) more significantly blocked tumor growth than saline as control. CTX/PLEL (i.t.) suppressed the tumor volume from 148.5 to 367.3 mm^3^ compared to saline as control, and the tumor inhibition rate was as high as 73.9%. Jevtana (i.t.) and Jevtana (i.v.) inhibited tumor growth for a few days after administration, but tumor growth was faster from day 10. On day 30, Jevtana (i.t.) and Jevtana (i.v.) only reduced the tumor volume to 927.2 and 705.8 mm^3^, respectively. These results are consistent with those of Figure 7B for tumors in vitro and in Figure 7E in terms of tumor weight. The mice treated with CTX/PLEL (i.t.) showed a low tumor weight, which was only 22.2% and 51.7% of the tumor weights from the mice treated with saline and Jevtana (i.v.), respectively. These results indicate that CTX loaded with temperature‐sensitive hydrogel PLEL can improve the antitumor activity of the drug in the treatment of colorectal cancer.

According to the average body weight change curves (Figure 7F) and HE staining of organs (Figure S1, Supporting Information), CTX/PLEL has excellent security, while Jevtana and Jevtana/PLEL have liver damage. In particular, in the intravenous Jevtana group, the weight of mice decreased dramatically from day 4, indicating systemic toxicity of Jevtana. In addition to drug toxicity, severe allergic reactions to Tween 80 injections, an excipient of Jevtana, have been reported in many patients.^[^ ^57^, ^58^ ^]^ CTX/PLEL (i.t.) is more favorable for clinical use than Jevtana/PLEL (i.t.). These results indicate that CTX/PLEL is a safe and effective anti‐tumor agent with great potential for future applications.

## 3 Conclusion

In this study, we used thermosensitive injectable hydrogel PLEL loaded with CTX to explore its anti‐tumor effect on mice with orthotopic colorectal cancer. CTX/PLEL is characterized by a solution state at room temperature and a hydrogel state at physiologic temperature. The excipients MPEG‐PCL and PDLLA‐PEG‐PDLLA have good biocompatibility and biodegradability. The simple material synthesis and preparation process renders this system cost‐effective and more conducive to clinical transformation. We established an orthotopic colorectal cancer model by transplantation subcutaneous tumors onto the cecum of mice. Compared with the classical orthotopic model of inoculating cells into the cecum wall, this method is more likely to reflect tumor metastasis and has a higher tumorigenesis rate. According to the results of experiments in vivo, CTX/PLEL significantly inhibited orthotopic colorectal cancer and liver metastasis in mice. We expect that this stable and hypotoxic drug delivery system with favorable anti‐tumor effects could provide an alternative and effective therapeutic treatment for colorectal cancer.

## 4 Experimental Section

### Materials

Cabazitaxel was purchased from Dalian Meilun Biotechnology Co., Ltd. Polyethylene glycol monomethyl ether (MPEG, MW = 2000), Poly (ethylene glycol) (PEG, Mn = 1500), 3‐(4, 5‐dimethylthiazol‐2yl) −2, 5‐diphenyl tetrazolium bromide, Sn(OCT)_2_, and acetonitrile were purchased from Sigma‐Aldrich Company in the United States; ε‐caprolactone and D, L‐Lactide was purchased from Alfa‐Aesar, CH_2_Cl_2_ was purchased from Tianjin Komil Chemical Reagent Co., Ltd, and petroleum ether was purchased from Tian jin Jinfeng Chemical Co., Ltd. Tween 80 and ethanol in water were purchased from Shanghai Aladdin Company; PBS (pH 7.4) was purchased from Chengdu Kelong Chemical Reagent Factory, China, and all reagents were analytically pure unless otherwise indicated.

The HCT‐116‐Luc (Luciferase‐expressing mouse colon carcinoma) cell line were obtained from the American Type Culture Collection (ATCC; Rockville, MD, USA). HCT‐1116‐Luc cells were grown in McCoy's 5A medium. The cell culture was maintained in a 37 °C incubator under a humidified 5% CO_2_ atmosphere.

Balb/c‐nu mice and balb/c mice used for antitumor tests were purchased from the HFK Bio‐Technology Co., Ltd., Beijing, China for the establishment of a colorectal cancer model and safety evaluation. All animal experiments were performed following the protocols approved by the Ethics Committee of the Animal Experimental Center of West China Hospital of Sichuan University. Approval number: 20 220 531 032.

### Synthesis of MPEG‐PCL and PDLLA‐PEG‐PDLLA (PLEL)

MPEG‐PCL was synthesized by ring‐opening polymerization of caprolactone (ε‐CL) initiated by polyethylene glycol monomethyl ether (MPEG) under the catalysis of stannous caprylate (Sn(OCT)_2_, 0.3%, w/w). PLEL was synthesized by ring‐opening polymerization of D, L‐lactide initiated by poly (ethylene glycol) (PEG) under the catalysis of Sn(OCT)_2_ (0.3%, w/w) (Figure S9, Supporting Information).

### Preparation and Characterization of CTX‐MPEG‐PCL Nanoparticles

CTX‐MPEG‐PCL nanoparticles were prepared using the emulsifying solvent evaporation method. Briefly, CTX and MPEG‐PCL were dissolved in acetone, thoroughly mixed, and then added to the deionized water containing 0.08% D‐α‐tocopheryl polyethylene glycol succinate drop by drop under magnetic stirring to form an oil‐in water emulsion. After magnetic stirring for 15 min, the emulsion was subjected to vacuum rotary evaporation at 37 °C to vaporize the acetone. An aqueous solution of nanoparticles loaded with CTX is formed. CTX‐MPEG‐PCL was filtered by 0.22 µm syringe filter (Millex‐LG, Millipore Co., USA) and stored at room temperature.

The particle size distribution and zeta potential of the nanoparticles were measured by dynamic laser scattering measurements using a Malvern Zetasizer Nano‐ZS (Zetasizer Nano ZSP, Malvern, England).^[^ ^59^, ^60^ ^]^ The test temperature was balanced 2 min before the test. The drug loading and encapsulation efficiency of CTX‐MPEG‐PCL nanoparticles were determined by HPLC (Agilent 1260 HPLC, USA).

### Preparation and Thermosensitive Sol‐Gel Phase Transition of CTX/PLEL

The PDLLA‐PEG‐PDLLA (L_1500_‐E_1500_‐L_1500_, PLEL) polymer powder was added into the CTX‐MPEG‐PCL nanoparticle solution and mixed by magnetic stirring to obtain homogeneous PLEL polymer solution. The concentration of PLEL is 15 wt.%. CTX/hydrogel was filtered by 0.22 µm syringe filter (Millex‐LG, Millipore Co., USA) and stored at 4 °C. The morphology of PLEL solution was observed by scanning electron microscope (SEM). CTX/PLEL was placed at room temperature (25 °C) to evaluate the stability of the preparation by macroscopic observation of the visible precipitate in the solution. This method was based on the Chinese Pharmacopeia 2010 (ChP, 2010). The morphological changes of CTX/PLEL at different temperatures were observed to investigate the thermosensitive sol‐gel phase transition.

The rheological behavior of CTX/PLEL during phase transition was measured by a rheometer (HAAKE Rheostress 6000, Thermo Scientific, USA). Briefly, the CTX/PLEL with a concentration of 15% was balanced in a 4°C refrigerator for 30 min. Then we detected the changes of the storage modulus (G′), loss modulus (G″), and viscosity (η) of CTX/PLEL hydrogel in the range of 18–60 °C at a heating rate of 1 °C min^−1^. To determine gelation time (t_gel_), G′ and G″ of CTX/PLEL hydrogel were observed when the temperature was controlled at 37 °C. Finally, the above experiments were repeated with blank PLEL solution to detect the modulus change of PLEL hydrogel after loading drugs.

The drug release of CTX/PLEL was quantitatively detected by high‐performance liquid chromatography (HPLC). First, 1 mL CTX/PLEL solution (15 wt.%) was equilibrated at 37 °C for 30 min, and then 10 mL of PBS buffer (pH 7.4) containing 0.1% w/v Tween 80 was slowly added to the gel and incubated at 37 °C under gentle shaking (100 rpm). At the set time points (2, 5.5, 8, 10, 24, 48, and 96 h), the release media was collected and replaced with fresh release media. The released CTX samples were quantified by HPLC. Acetonitrile/water (v/v, 7:3) was used as the mobile phase for all samples, and UV detection was performed at 232 nm.

### Safety Evaluation of CTX/PLEL In Vivo

To evaluate the in vivo safety of CTX/PLEL, we injected CTX/PLEL intraperitoneally into healthy balb/c mice at 8 mg kg^−1^. Orbital blood was taken on day 7 after administration for complete blood counts. The body weight of the mice was recorded every other day. Mice in control group were intraperitoneally injected with normal saline. Both groups received one injection. The mice were sacrificed on day 20.

### Antitumor Activity of CTX/PLEL In Vitro

The antitumor activity of CTX/PLEL in vitro was detected by MTT assay.HCT‐15 cells and HCT‐116 cells were cultured in 96‐well plates for 24 h and then added with different concentrations of CTX/PLEL. After incubation for 24(48) h, 20 ul MTT solution (5 mg mL^−1^ in saline) was added to each well. After 4 h, 150 uL DMSO was added to each well and oscillated for 10 min. The optical absorption of solution at 570 nm for each well was detected by microplate reader (Thermo MK3, USA).

### Orthotopic Mouse Model of Colorectal Cancer

Orthotopic tumor models can better reflect the disease development of colorectal cancer patients than the subcutaneous tumor model. There are two main methods to construct an orthotopic tumor model of colorectal cancer. One method is to inject tumor cells into the cecum wall. The other involves transplantation of a piece of subcutaneous tumor onto the cecum. Injection of a cell suspension into the cecal wall tends to lead to leakage post‐injection and low tumorigenesis rates, and the growth of tumors is less metastatic because the tumor cells in vitro are homogeneous. Transplantation of a piece of subcutaneous tumor has a higher rate of tumorigenesis and can grow a more heterogeneous population of cancer cells in vivo.^[^ ^61^, ^62^, ^63^, ^64^, ^65^, ^66^ ^]^ We established an orthotopic tumor model by transplantation of a piece of subcutaneous tumor onto the cecum of balb/c‐nu mice.

Colorectal cancer cells (HCT‐116‐luc) were injected subcutaneously into the right back of female balb/c‐nu mice aged 6–8 weeks. After two weeks, the subcutaneous tumor was removed, soaked in 75% alcohol, rinsed with normal saline three times, and cut into 2–3 mm pieces. Mice anesthetized with isoflurane were placed in a supine position on a sterile surgical darpe, applied 5% POvidone iodine solution on the abdomen, and cut open the abdomen to expose the cecum. The subcutaneous tumor was sutured to the cecum wall with a 6‐0 sized absorbable suture. The abdominal wall of the mice was closed with a 4‐0 sized suture. The mice were anesthetized by isoflurane throughout the operation and remained unconscious. To prevent infection, the mice were intramuscularly injected with penicillin solution on day 0,1,2 after surgery. In order to observe tumor development and metastasis, mice were injected intraperitoneally with D‐luciferin (15 mg mL^−1^, 200 uL) and bioluminescence was measured by a fluorescence imaging system (IVIS Lumina Series III, PerkinElmer, USA). After the mice were sacrificed, the whole intestine was collected for bioluminescent imaging.

### Anti‐Tumor Effects In Vivo: Anti‐Tumor Effects of CTX/PLEL in Orthotopic Mouse Model of Colorectal Cancer

Mice‐bearing orthotopic tumors were randomLy divided into four groups (4 mice in each group) and intraperitoneally injected with normal saline (i.p.), Jevtana (i.p., 8 mg kg^−1^ of CTX), CTX /PLEL (i.p., 4 mg kg^−1^ of CTX), or CTX/PLEL (i.p., 8 mg kg^−1^ of CTX). The four groups were single injection. Luminescence intensities were recorded every five days. The mice were sacrificed on day 20, and the whole intestine and tumor tissues were removed, photographed, and weighed.

### Anti‐Tumor Effects In Vivo: Anti‐Tumor Effects of CTX/PLEL in Subcutaneous Tumor Model of Colorectal Cancer

The in vivo antitumor activity of CTX/PLEL was further investigated by HCT‐15 human colorectal cancer subcutaneous tumor model. Colorectal cancer cells (HCT‐15) were injected subcutaneously (100 µL per mouse) at a density of 1 ×10^7^ cells mL^−1^ into the right back of female Balb/c‐nu mice aged 6–8 weeks. When the tumor volumes (*V*) reached to 100 mm^3^, the mice were randomLy divided into six groups (5 mice in each group) and injected with normal saline (i.t.), Jevtana (i.t., 2 mg kg^−1^ of CTX), CTX‐MPEG‐PCL NPs (i.t., 2 mg kg^−1^ of CTX), Jevtana (i.v., 15 mg kg^−1^ of CTX), Jevtana/PLEL (i.t., 2 mg kg^−1^ of CTX), or CTX/PLEL (i.t., 2 mg kg^−1^ of CTX). The intratumor groups were single injections, and the intravenous injection group was given on days 0, 3, and 6. The body weight and tumor volumes of the mice were recorded every other day. The mice were sacrificed on day 30, and the tumor tissues were removed, photographed, and weighed. The tumor inhibition rate was calculated using Equation.

(1) $$\;\mathrm{Tumor}\;\mathrm{inhibition}\;\mathrm{rate}\;\;\left(\%\right)=\frac{\;V_{control}-V_{sample}}{\;V_{control}}\;\;\times \;\;100\;\;\left(\%\right)$$

### Pathological Research

Collected heart, liver, spleen, lung, kidney, and tumor tissues were fixed in 10% formalin for two days. Hematoxylin and eosin staining (H&E) was performed on the heart, liver, spleen, lung, kidney, and tumor tissues in order to observe pathological changes of the mice. Tumor cell proliferation and apoptosis in mouse tumor tissues were observed by immunohistochemical staining of Ki‐67 (LabVision, MA, USA) and terminal deoxynucleotidyl transferase‐mediated nick‐end labeling (TUNEL, Promega, Madison, WI, USA) staining assays.

### Statistical Analysis

Statistical analysis was performed using SPSS software (International Business Machines Corporation, USA) and all of the results were expressed as mean ± SD. Student's *t*‐test or one‐way analysis of variance (ANOVA) was used for statistical analysis. The difference between the means was considered statistically significant at *p* < 0.05. The sample size (n) for each statistical analysis is indicated in detail in figure legends.

## Conflict of Interest

The authors declare no conflict of interest.

## Supporting information

Supporting Information

## Data Availability

Research data are not shared.
