# Supplementary material for: Cabazitaxel‐Loaded Thermosensitive Hydrogel System for Suppressed Orthotopic Colorectal Cancer and Liver Metastasis
Source: Adv Sci (Weinh). 2024 Jun 27;11(33):2404800. doi: 10.1002/advs.202404800 (PMC11434046; doi:10.1002/advs.202404800)
Supplement: Supplementary file 1 — Supporting Information [file ADVS-11-2404800-s001.docx]

***Supporting Information***

**Cabazitaxel-loaded Thermosensitive Hydrogel System for Suppressed Orthotopic Colorectal Cancer and Liver Metastasis**

Yu Chen, Liqun Dai, Kun Shi, Meng Pan, Liping Yuan, and Zhiyong Qian^*^

Department of Biotherapy, Cancer Center and State Key Laboratory of Biotherapy, West China Hospital, Sichuan University, Chengdu, 610041, China.

**Table S1.** The analysis of ^1^H-NMR spectrum of MPEG-PCL copolymers.

| **No.** | **Chemical shift (ppm)** | **Corresponding group** |
| --- | --- | --- |
| a | 3.37 | -C**H**_3_O- |
| b | 3.65 | -C**H**_2_C**H**_2_O- |
| c | 3.81 | -C**H**_2_CH_2_OCOCH_2_- |
| d | 4.22 | -CH_2_C**H**_2_OCOCH_2_- |
| e | 2.23 | -OCOC**H**_2_CH_2_CH_2_CH_2_CH_2_O- |
| f | 1.62 | -OCOCH_2_C**H**_2_CH_2_C**H**_2_CH_2_O- |
| g | 1.46 | -OCOCH_2_CH_2_C**H**_2_CH_2_CH_2_O- |
| h | 4.05 | -OCOCH_2_CH_2_CH_2_CH_2_C**H**_2_O- |

**Table S2.** The analysis of ^1^H-NMR spectrum of PDLLA-PEG-PDLLA copolymers.

| **No.** | **Chemical shift (ppm)** | **Corresponding group** |
| --- | --- | --- |
| a | 4.33 | -CH_2_C**H**_2_OCO- |
| b | 3.64 | -C**H**_2_C**H**_2_O- |
| c | 5.19 | -COC**H**(CH_3_)O- |
| d | 1.67 | -COCH(C**H**_3_)O- |


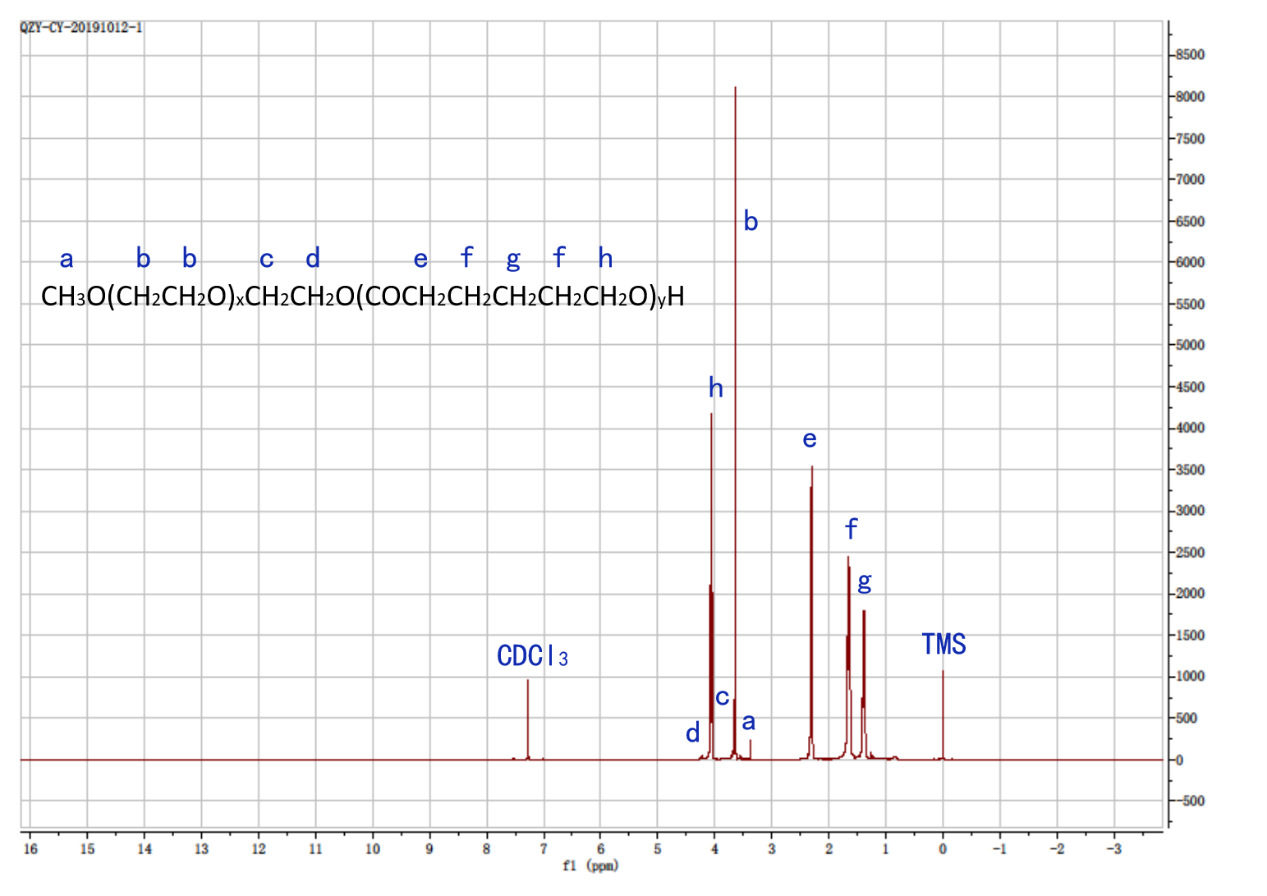


**Figure S1.** The ^1^H-NMR spectra of MPEG-PCL copolymers.


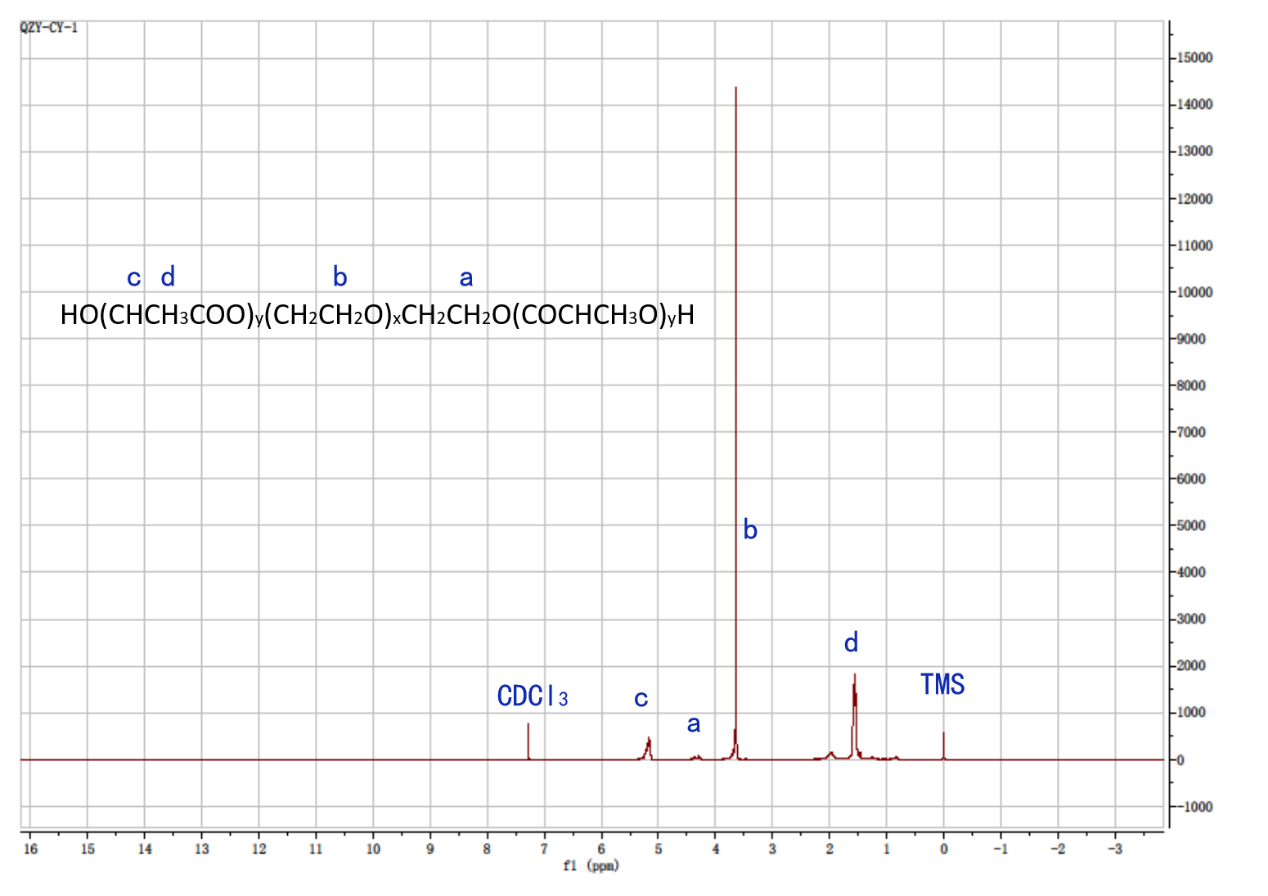


**Figure S2.** The ^1^H-NMR spectra of PDLLA-PEG-PDLLA copolymers.


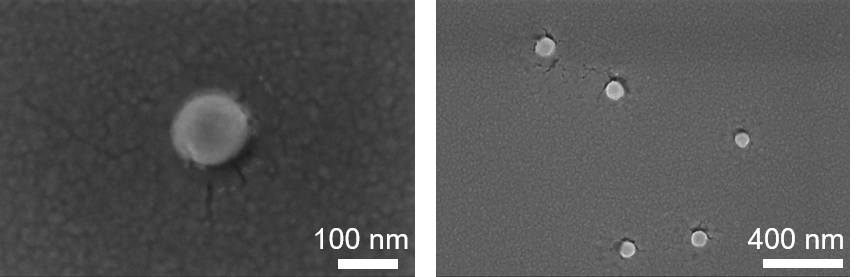


**Figure S3.** SEM images of PLEL micelles.


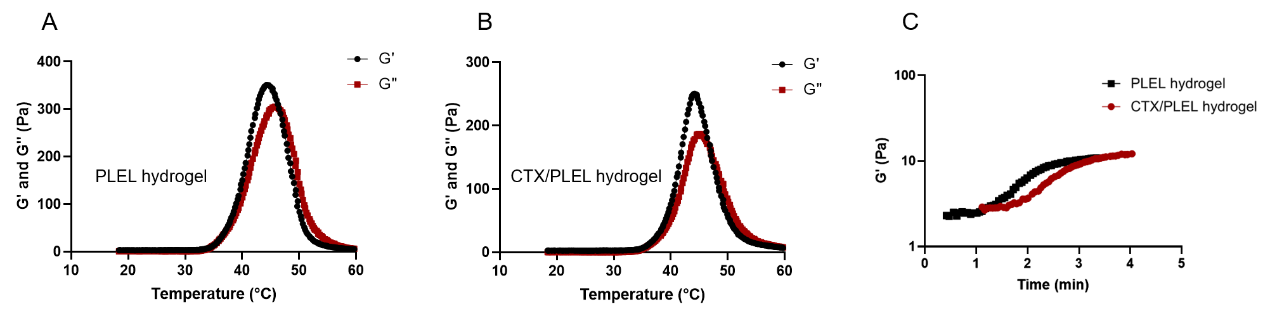


**Figure S4.** Rheological behavior for the CTX loaded PLEL hydrogel and PLEL hydrogel (A) Temperature-dependence of storage modulus (G') and loss modulus (G") for the PLEL hydrogel (15 wt%). (B)Temperature-dependence of storage modulus (G') and loss modulus (G") for the CTX loaded PLEL hydrogel (15 wt%). (C)Time-dependence of storage (G') for the CTX loaded PLEL hydrogel and PLEL hydrogel (15 wt%).


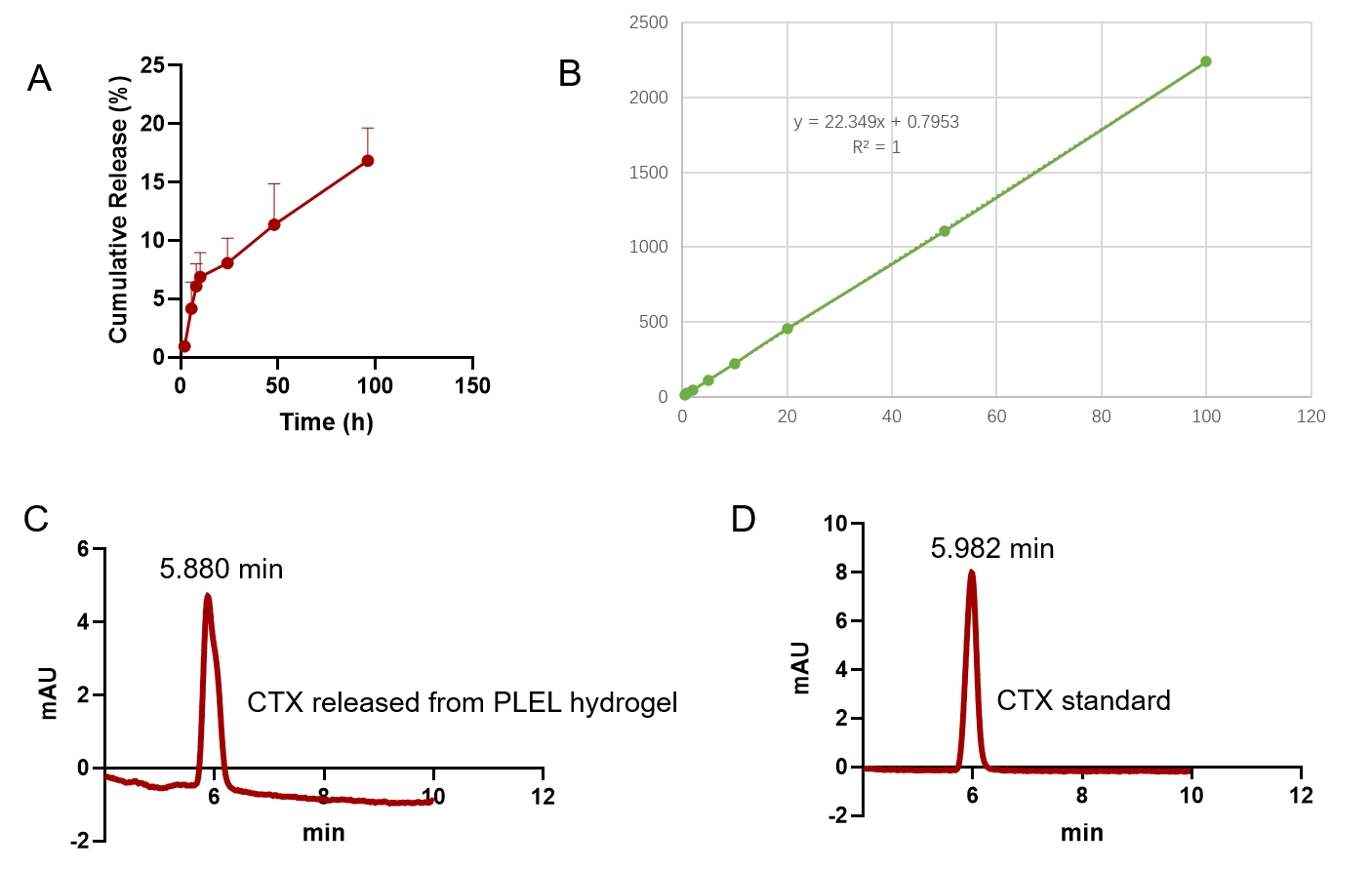


**Figure S5.** Pharmaceutical research on drug release. (A) *In vitro* drug release of CTX loaded PLEL hydrogel. (B) The standard curve of CTX detected by HPLC. (C) The HPLC chromatogram of CTX released from PLEL hydrogel. (D) The HPLC chromatogram of CTX standard. (n = 3).


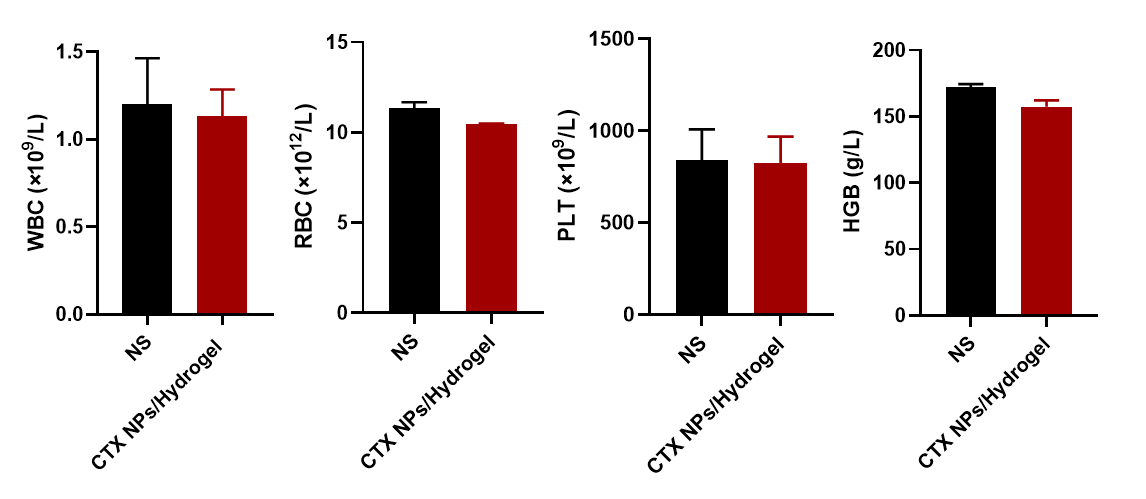


**Figure S6.** Complete blood counts of balb/c mice after intraperitoneal injection at 7 days, including white blood cells (WBC), red blood cells (RBC), platelets (PLT), and hemoglobin (HGB). (n = 3).


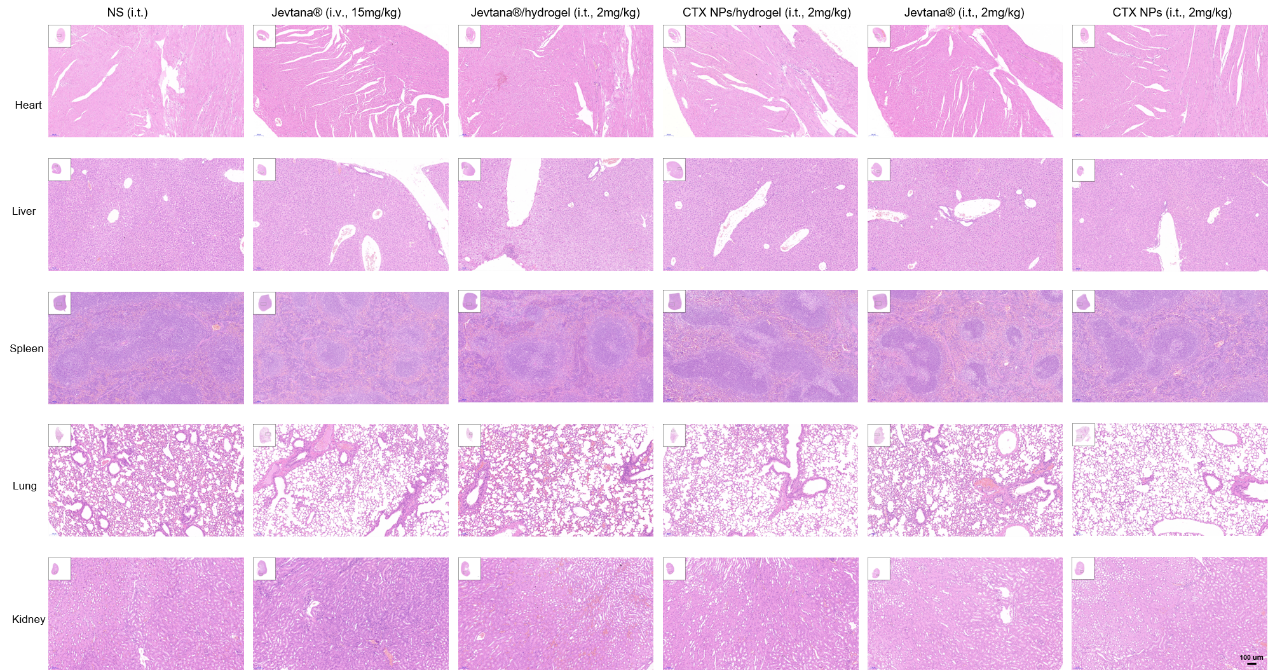


**Figure S7.** H&E-staining of the heart, liver, spleen, lung and kidney tissues of each group of subcutaneous tumor-bearing mice after treatment. Scale bar:100 μm.


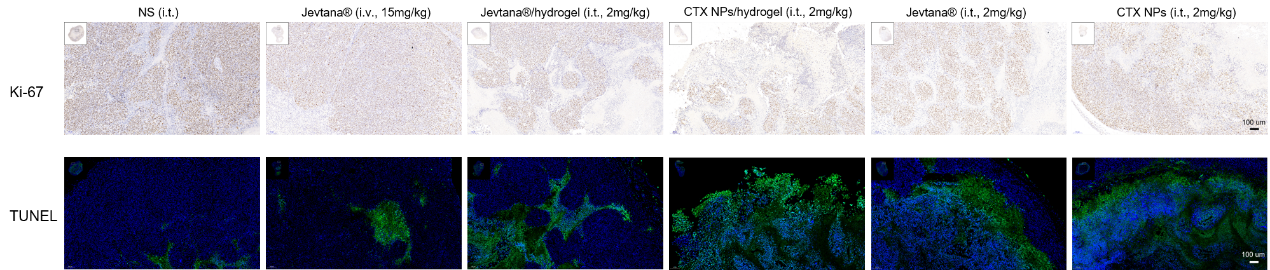


**Figure S8.** Histological analysis of subcutaneous tumor slices after Ki-67 staining and TUNEL assay. Scale bar:100 μm.


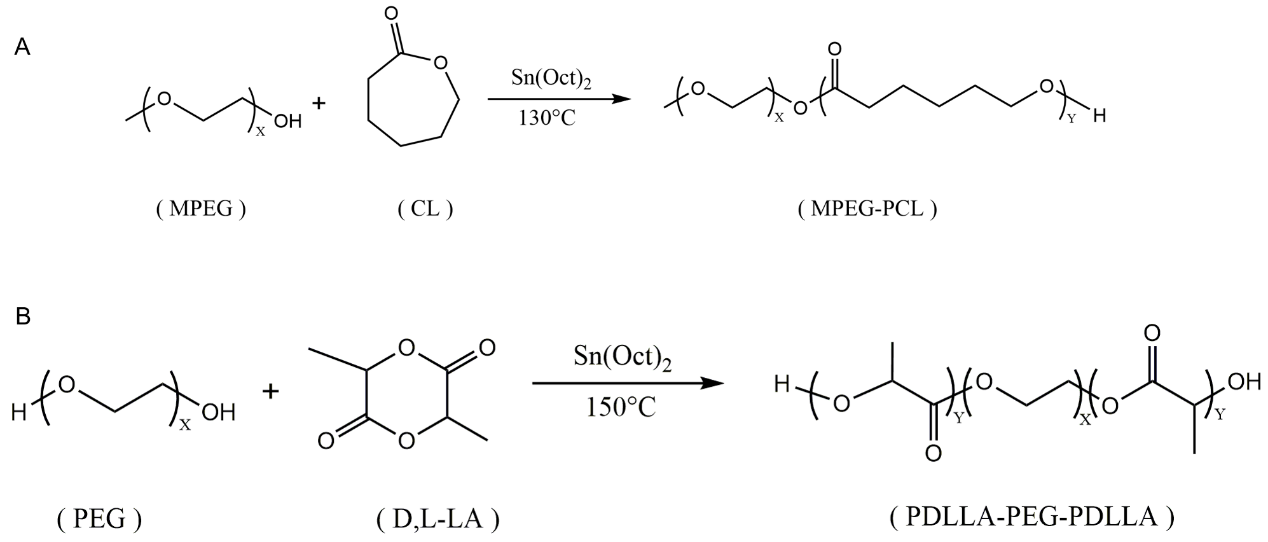


**Figure S9.** Synthetic schematic diagram of MPEG-PCL (A) and PDLLA-PEG-PDLLA (B) copolymers.
